# MicroRNAs as the critical regulators of Cisplatin resistance in ovarian cancer cells

**DOI:** 10.1186/s13048-021-00882-1

**Published:** 2021-09-30

**Authors:** Meysam Moghbeli

**Affiliations:** Department of Medical Genetics and Molecular Medicine, School of Medicine, Mashhad University of Medical Sciences, Mashhad, Iran

**Keywords:** Ovarian cancer, Chemo-resistance, Cisplatin, MicroRNA, Chemotherapy

## Abstract

**Background:**

Ovarian cancer (OC) is one of the leading causes of cancer related deaths among women. Due to the asymptomatic tumor progression and lack of efficient screening methods, majority of OC patients are diagnosed in advanced tumor stages. A combination of surgical resection and platinum based-therapy is the common treatment option for advanced OC patients. However, tumor relapse is observed in about 70% of cases due to the treatment failure. Cisplatin is widely used as an efficient first-line treatment option for OC; however cisplatin resistance is observed in a noticeable ratio of cases. Regarding, the severe cisplatin side effects, it is required to clarify the molecular biology of cisplatin resistance to improve the clinical outcomes of OC patients. Cisplatin resistance in OC is associated with abnormal drug transportation, increased detoxification, abnormal apoptosis, and abnormal DNA repair ability. MicroRNAs (miRNAs) are critical factors involved in cell proliferation, apoptosis, and chemo resistance. MiRNAs as non-invasive and more stable factors compared with mRNAs, can be introduced as efficient markers of cisplatin response in OC patients.

**Main body:**

In present review, we have summarized all of the miRNAs that have been associated with cisplatin resistance in OC. We also categorized the miRNAs based on their targets to clarify their probable molecular mechanisms during cisplatin resistance in ovarian tumor cells.

**Conclusions:**

It was observed that miRNAs mainly exert their role in cisplatin response through regulation of apoptosis, signaling pathways, and transcription factors in OC cells. This review highlighted the miRNAs as important regulators of cisplatin response in ovarian tumor cells. Moreover, present review paves the way of suggesting a non-invasive panel of prediction markers for cisplatin response among OC patients.

## Background

Ovarian cancer (OC) is the most common cause of cancer related deaths among females [1]. Histopathological classification categorizes the OC into germ cell, sex cord stromal, and epithelial tumors among them the epithelial ovarian cancer (EOC) is the most common type (90% of cases) with the highest rate of occurrence and mortality [2]. There are various risk factors associated with OC including continuous ovulation, increased gonadotropins exposure, and inflammatory cytokines [3, 4]. There is a poor prognosis in advanced stage OC tumors with a 5-year survival rate of less than 20% [5]. Majority of OC patients are diagnosed in advanced stages (survival rate of below 45%), due to the asymptomatic tumor progression and lack of efficient screening methods [6]. A combination of surgical resection and platinum based-therapy is the common treatment option for advanced OC patients. However, tumor relapse is observed in about 70% of cases due to the treatment failure [7]. Cisplatin or platinum diamminodichloride (DDP) is widely used as an efficient first-line treatment option for OC; however there is DDP resistance in a noticeable ratio of cases [8, 9]. DDP exerts its therapeutic role by forming DNA adducts which results in deregulation of DNA replication and transcription [10]. About 80% of OC patients are DDP sensitive; however there is a high ratio of cisplatin resistance mediated mortality among them in a few years [11]. Various cellular processes including drug-efflux, detoxification, DNA repair, apoptosis, autophagy, signaling pathways, and microRNAs (miRNAs) are involved in chemo resistance of cancer cells [12–14]. DDP resistance in OC is associated with abnormal drug transportation, increased detoxification, abnormal apoptosis, and abnormal DNA repair ability [15, 16]. MiRNAs are a class of endogenous non-coding RNAs that have a critical role in gene expression regulation via mRNA degradation and translational inhibition of their target genes [17]. They are critical factors involved in cell proliferation, apoptosis, and differentiation [18]. It has been shown that miRNAs are involved in platinum drug-resistance and prognosis in OC [19]. MiRNAs maturation is a multi-step molecular post transcriptional process that is initiated by Drosha/DGCR8 in the nucleus and continued by Dicer in cytoplasm. Reduced levels of Dicer expression has been significantly correlated with advanced tumor stage and poor prognosis among EOC patients [20]. It has been reported that *miR-98-5p* significantly increased DDP resistance via DICER1 targeting and general miRNA down regulation among EOC patients. *MiR-98-5p* exerted its role in induction of DDP resistance by *miR-152* down regulation following the DICER1 targeting in EOC [21]. Regarding, the lack of efficient method to distinguish DDP resistant from sensitive, it is required to clarify the molecular mechanisms involved in DDP resistance to provide novel efficient therapeutic modalities to improve the clinical outcomes of OC patients. MicroRNAs as non-invasive and more stable factors compared with mRNAs, can be introduced as efficient markers of DDP response in OC patients. In present review, we have summarized all of the miRNAs that have been reported to be associated with DDP resistance in OC (Table 1). MiRNAs were also categorized based on their targets to clarify their probable molecular mechanisms during DDP resistance in ovarian tumor cells.

**Table 1:** All of the miRNAs associated with DDP resistance in ovarian cancer

Study	Year	Gene	Country	Target	Samples	Results
APOPTOSIS						
Chen [22]	2017	miR-509-3p	China	BCL2	SKOV3 and OVCAR3 cell lines	Increased DDP sensitivity.
Li [23]	2019	miR-142-5p	China	XIAP	19 patients SKOV3 and OVCAR3 cell lines	Increased DDP sensitivity.
Li [24]	2017	miR-146a-5p	China	XIAP, BCL2L2, and BIRC5	OVCAR3 and SKOV3 cell lines	Increased DDP sensitivity.
Chen [25]	2016	miR-509-3p	China	XIAP	SKOV3 and A2780 cell lines	Increased DDP sensitivity.
Pang [26]	2014	miR-519d	China	XIAP	7 patients A2780, SKOV3, and OVCAR3 cell lines	Increased DDP sensitivity.
Zhang [27]	2013	miR-130a	China	XIAP	A2780 cell line	Increased DDP sensitivity.
Chen [28]	2016	miR-155	China	XIAP	SKOV3 and A2780 cell lines	Increased DDP sensitivity.
Liu [29]	2018	miR-335-5p	China	BCL2L2	OV90m A2780m and OVCAr3 cell lines	Increased DDP sensitivity.
Wang [30]	2020	miR-454	China	BIRC5	A2780 and SKOV3 cell lines	Increased DDP sensitivity.
Rao [31]	2013	miR-106a	China	MCL1	A2780 cell line	Increased DDP sensitivity.
Chen [32]	2020	miR-137	China	MCL1	OVCAR3 cell line	Increased DDP sensitivity.
Su [33]	2019	miR-142-5p	China	MCL1	OVCAR3 and SKOV3 cell lines	Increased DDP resistance.
Zuo [34]	2020	miR-34a-5p	China	PDL1	SKOV3 and A2780 cell lines	Increased DDP sensitivity.
Bieg [35]	2019	miR-424-3p	Poland	LGALS3	TOV21G and SKOV3 cell lines	Increased DDP sensitivity.
Kong [36]	2011	miR-125b	China	BAK1	OV2008 cell line	Increased DDP resistance.
Echevarria-vargas [37]	2014	miR-21	Puerto Rico	PDCD4	A2780 and SKOV3 cell lines	Increased DDP resistance.
Li [38]	2014	miR-106a	China	PDCD4	OVCAR3 cell line	Increased DDP resistance.
Wambecke [39]	2021	miR-27a-5p	France	UBE2N	3 patients OAW42 and OVCAR3 cell lines	Increased DDP sensitivity.
DNA repair and cell cycle regulation						
Liu [40]	2017	miR-216b	China	PARP1	51 patients SKOV3 cell line	Increased DDP sensitivity.
Zhu [41]	2020	miR-770-5p	China	PARP1	19 patients A2780 and SKOV3 cell lines	Increased DDP sensitivity.
Sun [42]	2013	miR-9	China	BRCA1	113 patients A2780 and OV2008 cell lines	Increased DDP sensitivity.
Zhao [43]	2016	miR-770-5p	China	ERCC2	86 patients A2780 and C13 cell lines	Increased DDP sensitivity.
Guo [44]	2019	miR-98-5p	China	CDKN1A	42 patients SKOV3 and A2780 cell lines	Increased DDP resistance.
Guo [45]	2016	miR-100	China	mTOR and PLK1	SKOV3 cell line	Increased DDP sensitivity.
Cheng [46]	2018	miR-409-3p	China	FIP200	13 patients SKOV3, OVCAR3, CAOV3, COC1, and OV1063 cell lines	Increased DDP sensitivity.
Signaling pathways						
Wu [47]	2018	miR-503	China	PI3K	SKOV3 cell line	Increased DDP sensitivity.
Qin [48]	2017	miR-708	China	IGF2BP1	A2780 and SKOV3 cell lines	Increased DDP sensitivity.
Shi [49]	2018	miR-205-5p	China	PTEN	OV2008 cell line	Increased DDP resistance.
Fu [50]	2012	miR-93	China	PTEN	OVCAR3 and SKOV3 cell lines	Increased DDP resistance.
Li [51]	2021	miR-106a	China	PTEN	12 patients SKOV3 cell line	Increased DDP resistance.
Yang [52]	2020	miR-34c	China	MET	25 patients A2780 and SKOV3 cell lines	Increased DDP sensitivity.
Zhang [53]	2018	miR-1294	China	IGF1R	30 patients SKOV3 cell line	Increased DDP resistance.
Wang [54]	2013	miR-199a	China	mTOR	OV2008 cell line	Increased DDP sensitivity.
Chen [55]	2019	miR-1271	China	mTOR	SKOV3 cell line	Increased DDP sensitivity.
Xu [56]	2015	miR-497	China	mTOR and p70S6K1	41 patients SKOV3 and A2780 cell lines	Increased DDP sensitivity.
Zhang [57]	2020	miR-548e	China	CXCR4	17 patients CAOV3, OVCAR3, and SKOV3 cell lines	Increased DDP sensitivity.
Van jaarsveld [58]	2015	miR-634	Netherlands	MAPK	A2780, OV56, OAW42, TOV112D, and TOV21G cell lines	Increased DDP sensitivity.
Jiang [59]	2021	miR-7	China	ERK	6 patients SKOV3 cell line	Increased DDP resistance.
Xu [60]	2018	miR-378a-3p	China	MAPK1	62 patients OVCAR3 and SKOV3 cell lines	Increased DDP sensitivity.
Zhao [61]	2014	miR-224-5p	China	PRKCD	41 patients OV2008 and A2780 cell lines	Increased DDP resistance.
Zhou [62]	2014	miR-449a	China	NOTCH1	SKOV3 and A2780 cell lines	Increased DDP sensitivity.
Niu [63]	2019	miR-338-3p	China	WNT2B	54 patients SKOV3 and A2780 cell lines	Increased DDP sensitivity.
Dai [64]	2019	miR-195-5p	China	PSAT1	77 patients A2780, SKOV3, and HO8910 cell lines	Increased DDP sensitivity.
Transcription factors and methylation						
Xiao [65]	2019	miR-34c	China	SOX9	54 patients CAOV3, OVCAR3, SKOV3, and A2780 cell lines	Increased DDP sensitivity.
Jia [66]	2021	miR-491-5p	China	SOX3	90 patients HO8910, A2780, SKOV3, and CAOV3 cell lines	Increased DDP resistance.
Zhang [67]	2020	miR-21	China	C-MYB	ES2 and OVCAR3 cell lines	Increased DDP sensitivity.
Jiang [68]	2018	miR-139-5p	China	C-JUN	SKOV3 and A2780 cell lines	Increased DDP sensitivity.
Li [69]	2019	miR-143	China	FOSL2	56 patients SKOV3 and A2780 cell lines	Increased DDP sensitivity.
Jin [70]	2019	miR-210-3p	China	E2F3	SKOV3 cell line	Increased DDP sensitivity.
Sun [71]	2019	miR-137	USA	EZH2	PEO1, PEO4, OV90, and IGROV1 cell lines	Increased DDP sensitivity.
Zhu [72]	2016	miR-186	China	TWIST1	52 patients A2780, OV2008, OVCAR3, SKOV3, and CAOV3 cell lines	Increased DDP sensitivity.
Cao [73]	2018	miR-363	China	SNAIL	107 patients A2780 and OV2008 cell lines	Increased DDP sensitivity.
Zhang [74]	2019	miR-132	China	BMI1	SKOV3 cell line	Increased DDP sensitivity.
Dong [75]	2021	miR-205	China	ZEB2	A2780 and SKOV3 cell lines	Increased DDP sensitivity.
Liu [76]	2014	miR-101	China	EZH2	70 patients A2780 and SKOV3 cell lines	Increased DDP sensitivity.
Zhang [77]	2020	miR-138-5p	China	EZH2 and SIRT1	A2780 and SKOV3 cell lines	Increased DDP sensitivity.
Chen [78]	2018	miR-139-5p	China	RNF2	66 patients A2780 cell line	Increased DDP sensitivity.
Feng [79]	2017	miR-199a	China	HIF1a	23 patients OV2008 cell line	Increased DDP sensitivity.
Han [80]	2017	miR-30-5p	China	DNMT1	A2780 cell line	Increased DDP sensitivity.
Xiang [81]	2014	miR-152 and miR-185	China	DNMT1	SKOV3 and A2780 cell lines	Increased DDP sensitivity.
Liu [82]	2019	miR-200b/c	China	DNMT3A, DNMT3B, and SP1	93 patients SKOV3 and A2780 cell lines	Increased DDP sensitivity.
Transporters and structural factors						
Sun [83]	2015	miR-186	China	MDR1	OVCAR and A2780 cell lines	Increased DDP sensitivity.
Tian [84]	2016	miR-595	China	ABCB1	35 patients HG-SOC, HO8910, SKOV3, and ES2 cell lines	Increased DDP sensitivity.
Yang [85]	2012	miR-130a	China	ABCB1	SKOV3 cell line	Increased DDP sensitivity.
Wu [86]	2016	miR-873	China	ABCB1	A2780 and OVCAR3 cell lines	Increased DDP sensitivity.
Xiao [87]	2018	miR-514	China	ABCA1, ABCA10, and ABCF2	SKOV3 and OVCA433 cell lines	Increased DDP sensitivity.
Wu [88]	2020	miR-194-5p	China	SLC40A1	A2780 and COC1 cell lines	Increased DDP resistance.
Xiao [89]	2018	miR-139	China	ATP7A/B	37 patients CAOV3 and SNU119 cell lines	Increased DDP sensitivity.
Yu [90]	2014	miR-29	China	COL1A1	A2780 and SKOV3 cell lines	Increased DDP sensitivity.
Cui [91]	2018	miR-199a-3p	China	ITGB8	58 patients SKOV3 cell line	Increased DDP sensitivity.
Wu [92]	2021	miR-139-5p	China	SDC4	30 patients A2780 cell line	Increased DDP resistance.
Ding [93]	2021	miR-138-5p	China	SDC3	42 patients EB0405, CAOV3, and SKOV3 cell lines	Increased DDP sensitivity.
Han [94]	2021	miR-1305	China	CNTN1	70 patients SKOV3 and A2780 cell lines	Increased DDP sensitivity.
Samuel [95]	2016	miR-31	UK	KCNMA1	A2780 and OVCAR5 cell lines	Increased DDP resistance.
Van jaarsveld [96]	2013	miR-141	Netherlands	KEAP1	132 patients A2780, OV56, OAW42, TOV112D, and TOV21G cell lines	Increased DDP resistance.

## Main text

### Apoptosis

**Fig. 1:**
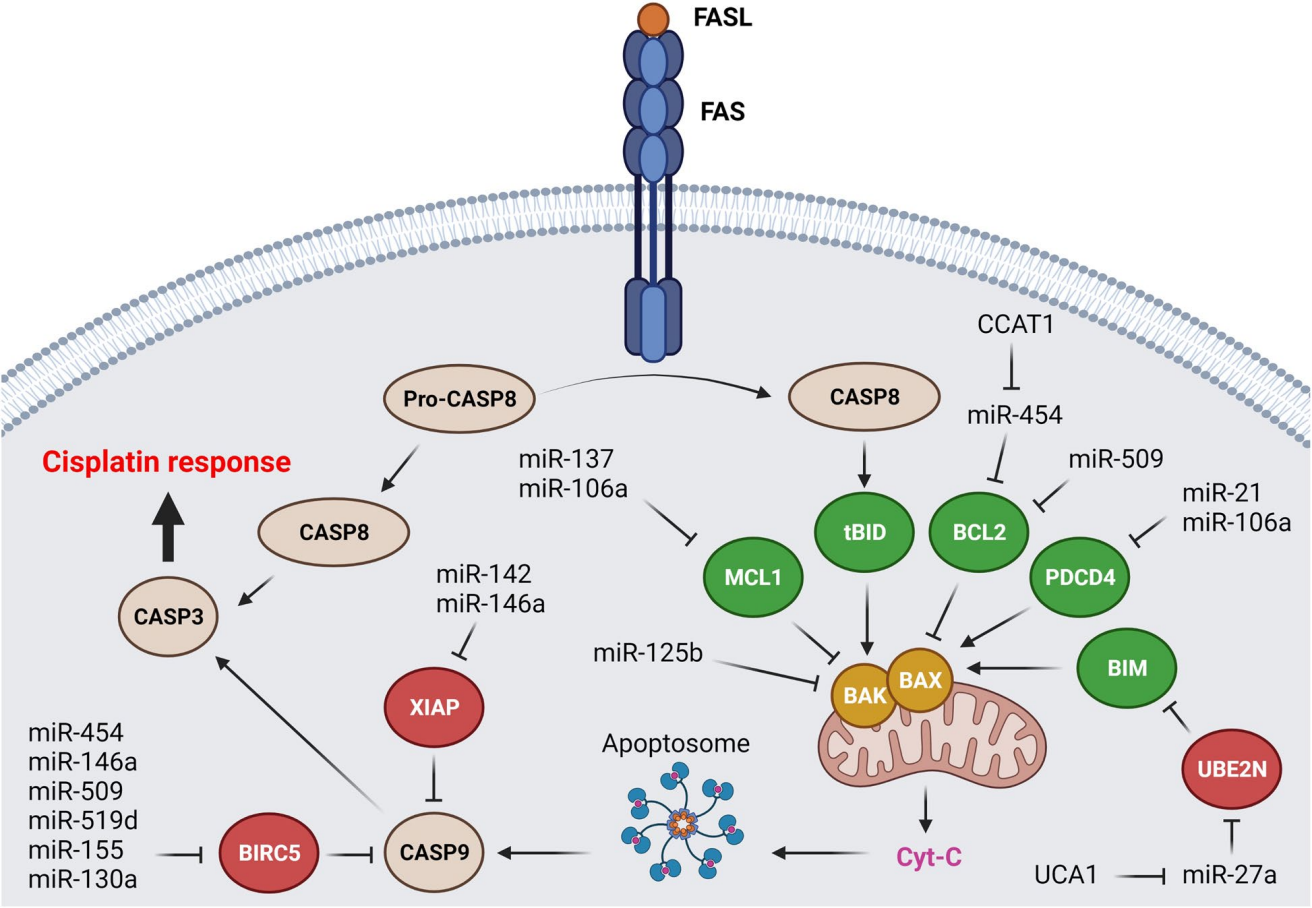
MicroRNAs are involved in regulation of DDP-mediated apoptosis in ovarian tumor cells

MicroRNAs have critical roles in regulation of DDP-mediated apoptosis in ovarian tumor cells (Fig. 1). Inhibitors of apoptosis proteins (IAPs) are involved in regulation of cell proliferation, motility, and death [97]. XIAP and BIRC3 are important members of IAP family associated with tumor progression via caspase suppression in different tumors [98, 99]. BCL2 is a family of proteins involved in positive or negative regulation of intrinsic mitochondrial apoptosis pathways in response to physiological and cytotoxic agents [100]. It has been observed that *miR-509-3p* induced DDP sensitivity in OC cells by targeting BCL2 [22]. BCL2L2 and BIRC5 are anti-apoptotic members of BCL2 and IAP families, respectively [101, 102]. It has been shown that *miR-142-5p* increased DDP response by XIAP targeting in OC. There was a converse association between the levels of *miR-142-5p* and XIAP expressions in OC patients [23]. *MiR-146a-5p* also induced DDP-mediated apoptosis by XIAP, BCL2L2, and BIRC5 targeting in OC cells [24]. It has been reported that *miR-509-3p*, *miR-519d*, *miR-155*, and *miR-130a* promoted DDP mediated apoptosis through XIAP targeting in OC cells [25–28]. Another group has been reported that *miR-335-5p* increased the DDP sensitivity in OC cells via BCL2L2 targeting. *MiR-335-5p* also reduced DDP resistance and ovarian tumor growth in nude mice [29]. Long non coding RNAs (LncRNAs) are a class of non-coding RNAs with critical regulatory functions on miRNAs and mRNAs as competing endogenous RNA (ceRNA) via sponging. Colon cancer-associated transcript 1 (CCAT1) is considered as an oncogenic lncRNA in colorectal cancer [103]. *CCAT1* up regulation has been observed in DDP-resistant OC cells. *CCAT1* was involved in DDP response of OC cells via regulation of apoptosis-related proteins in which it down regulated the BCL-2 and BIRC5, while induced BAX. *CCAT1* conferred DDP resistance by regulation of BIRC5 via *miR-454* sponging in OC cells [30]. MCL-1 is an anti-apoptotic member of BCL-2 family that is involved in cell survival and resistance toward chemotherapeutic mediated apoptosis. It is associated with DDP and paclitaxel resistance in OC [104, 105]. It has been shown that there were *miR-106a* and *miR-137* down regulations in DDP-resistant OC cell line. They increased DDP sensitivity via MCL-1 targeting in ovarian tumor cells [31, 32]. Neurofibromatosis type 1 (NF1) is a tumor suppressor involved in regulation of PIK3*/*AKT*/*mTOR and MAPK signaling pathways by Ras inactivation [106–108]. Loss of NF1 can also inhibit ZNF423 transcription factor that results in activation of EMT-related transcriptional factors [109, 110]. NF1 knockdown increased OC cells resistance to DDP-mediated apoptosis through MCL1 inhibition via *miR-142-5p* [33]. Immune escape mediated by cytotoxic T lymphocytes (CTL) dysfunction can be a critical reason of chemo resistance [111]. Majority of tumor cells are commonly eliminated by chemotherapy and immune system is also responsible for remaining tumor cells elimination. However, tumor cells escaping from chemotherapy can obtain immune-tolerance via CTL dysfunction [112, 113]. Programmed cell death 1 (PD-1) is one of the main negative regulators of T cell activation [114, 115]. PD-L1 produced by tumor cells is associated with stimulation of CTL apoptosis during tumor cells immune tolerance [116]. It has been shown that there were higher levels of PD-L1 expressions in DDP-resistant cells compared with parental. *MiR-145* was also down regulated following the DDP treatment which was associated with PD-L1 up regulation in OC cells [117]. PD-L1 up regulation and *miR-34a-5p* down regulation have been also observed in DDP-resistant OC cells which suggested *miR-34a-5p* as regulator of DDP response via PD-L1 targeting [34]. Galectin-3 (LGALS3) belongs to the lectin family of proteins involved in cell adhesion and angiogenesis. It is also involved in apoptosis via regulation of BCL-2 [118, 119]. It has been reported that *miR-424-3p* increased DDP response by LGALS3 targeting in OC cells [35]. BAK1 is a pro-apoptotic member of BCL2 family that is located in mitochondrial membrane and involved in cytochrome c release during intrinsic apoptosis pathway. It has been reported that there was *miR-125b* up regulation in DDP-resistant OC cells. *MiR-125b* induced DDP resistance via BAK1 targeting in OC cells [36]. *PDCD4* is a tumor suppressor that induces the apoptosis via activation of *BAX* followed by the mitochondrial cytochrome C release [120]. JNK-1/c-Jun pathway up regulated *miR-21* in DDP resistant OC cells that reduced PDCD4 levels [37]. Another study showed that *miR-106a* was up regulated in DDP-resistant compared with sensitive OC cell lines. *MiR-106a* regulated DDP resistance via PDCD4 targeting [38]. UBE2N is an ubiquitin ligase involved in BIM degradation. It has been reported that UCA1 down regulation sensitized OC cells to DDP through *miR-27a-5p* up regulation that results in UBE2N inhibition. Subsequently, BIM as a proapoptotic factor promotes DDP sensitivity in OC cells. Therefore, UCA1/miR-27a-5p/UBE2N axis can regulate DDP response in OC cells via BIM [39].

### DNA repair and cell cycle regulation

Majority of chemotherapeutic agents act through DNA damages. However, tumor cells can overcome to such DNA damages via activation of repair mechanisms [121]. PARP1 is involved in base excision repair by interaction with DNA protein kinase subunit to assemble all required proteins during double-strand breaks repair [122, 123]. It has also an important role in genetic stability via induction of homologous recombination (HR) [124]. It has been reported that there was significant *miR-216b* down regulation in DDP-resistant OC cell line compared with sensitive cells. *MiR-216b* significantly increased DDP sensitivity via PARP-1 targeting in OC cells [40]. NEAT1 is an oncogenic lncRNA in ovarian tumor cells. NEAT1 down regulation reduced DDP resistance and induced apoptosis via *miR-770-5p* sponging to regulate PARP1 in OC cells [41]. BRCA1 is also an important component of HR repair pathway [125, 126]. It has been shown that there was an inverse association between *miR-9* and BRCA1 expressions in OC cell lines and tissues. *MiR-9* increased DDP efficiency by BRCA1 targeting in OC [42]. ERCC2 as one of the components of nucleotide excision repair (NER) is involved in DNA replication and repair. Since, DDP activity is mediated by the formation of DNA adducts, NER repair system can increase DDP resistance via adducts removal [127, 128]. A converse association has been observed between *miR-770-5p* and ERCC2 expressions in OC patients with DDP chemotherapy in which *miR-770-5p* increased DDP sensitivity through ERCC2 inhibition [43]. Cancer-associated fibroblasts (CAFs) are a group of stromal cells that cause poor prognosis in OC patients [129]. They are involved in tumor progression and chemo resistance by secretion of exosomes in tumor microenvironment [130]. Cyclin-dependent kinase inhibitor 1A (CDKN1A) is a member of Cip/Kip family of cell cycle regulators [131]. It has been reported that CAF-derived exosomes with *miR-98-5p* were associated with DDP resistance in OC via CDKN1A inhibition. There was also higher levels of CDKN1A expressions in DDP-sensitive compared with DDP resistant OC cells [44]. mTOR and PLK1 belong to serine/threonine kinase family that are involved in cell proliferation, metabolism, and invasion [132]. PLK1 regulates the cell cycle by CDC25C activation that activates the cyclinB/CDC2 complex. It also activates the anaphase-promoting complex (APC) to maintain connection of sister chromatids. There were significant mTOR and PLK1 over expressions in SKOV3/DDP cells compared with SKOV3 cells. *MiR-100* increased DDP sensitivity by mTOR and PLK1 targeting in OC cells [45]. Fip200 is a critical factor involved in DNA repair following ionizing radiation mediated damage. It is also a positive regulator of RB1 that prevents the G1 to S phase progression during the cell cycle. *MiR-409-3p* suppressed the autophagy mediated by Fip200 that resulted in increased DDP sensitivity in OC cells [46].

### Signaling pathways

**Fig. 2:**
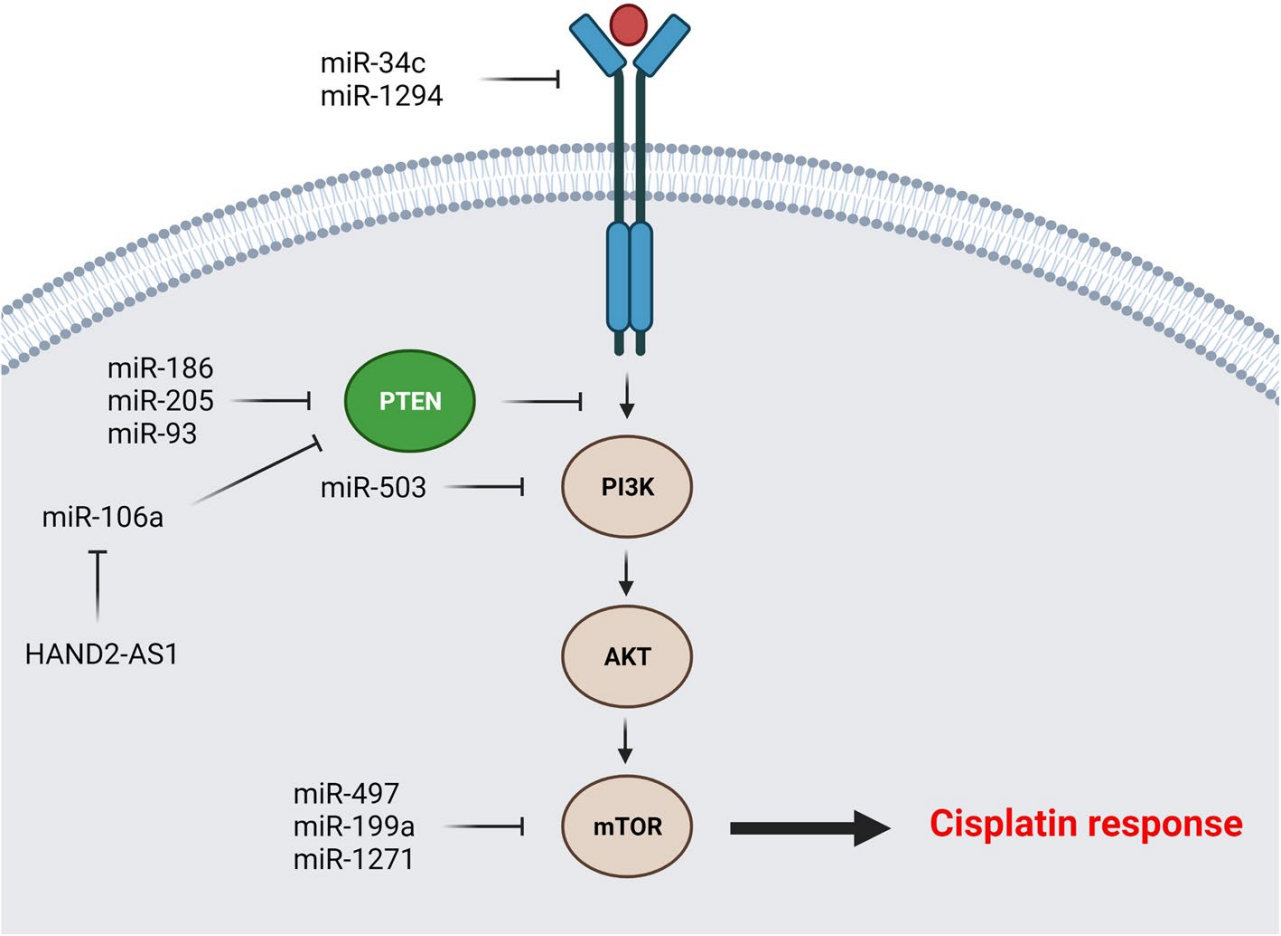
MicroRNAs are involved in regulation of DDP response via the PI3K/AKT pathway in ovarian tumor cells

PI3K/AKT is a pivotal signaling pathway involved in regulation of various cellular processes such as cell proliferation and apoptosis [133]. This signaling pathway has also a critical role in DDP sensitivity of tumor cells [134]. Therefore, miRNAs can regulate the DDP response in ovarian tumor cells via PI3K/AKT pathway (Fig. 2). PI3K p85 is the stabilizer and regulatory subunit of PIK3CA [135]. Apoptotic resistance is an evasion mechanism used by tumor cells for drug resistance. Increased activity of PI3K is associated with suppression of DDP mediated apoptosis in tumor cells [136]. PI3K/AKT pathway induces MDM2 phosphorylation and nucleus translocation, where it directly inhibits p53 to induce drug resistance [137]. AKT can also phosphorylate and inactivate the BAD pro-apoptotic factor [138]. It has been reported that there was a significant *miR-503* down regulation in DDP-resistant OC cell lines in comparison with parental. *MiR-503* down regulated the PI3K via PI3K p85 targeting which resulted in increased DDP sensitivity in OC cells [47]. IGF2BP1 is an oncogenic member of RNA-binding IGF2BP protein family [139]. It has been reported that there was *miR-708* down regulation in DDP-resistant OC cells compared with parental controls. *MiR-708* significantly increased the CASP-3 cleavage in DDP-resistant OC cells following DDP treatment which resulted in chemo sensitization via apoptosis induction. Moreover, *miR-708* sensitized the OC cells toward the DDP via IGF2BP1 targeting and AKT inhibition [48]. AKT is the key effector of PI3K signaling that is negatively regulated by phosphatase and tensin homolog (PTEN). It is involved in cell proliferation and metabolism via its downstream effectors such as GSK3, mTORC1, and FOXO [140]. It has been reported that *miR-186* regulated DDP sensitivity through PIK3R3 and PTEN targeting while APAF1 induction in OC cells [141]. *MiR-205-5p* up regulation has been observed in DDP-resistant OC cells. *MiR-205-5p* was associated with DDP resistance through inhibition of PTEN/AKT pathway in OC. Suppression of *miR-205-5p* increased the levels of PTEN expression that attenuated the p-AKT [49]. *MiR-93* also induced DDP resistance via PTEN down regulation in OC cells. Moreover, *miR-93* induced AKT1 phosphorylation which increased cell survival and suppressed apoptosis [50]. It has been reported that HAND2-AS1 promoted cell apoptosis through miR-106a/PTEN axis in SKOV3/DDP cells [51]. MET belongs to the tyrosine kinase receptors that functions as an oncogenic factor [142]. Activation of MET signaling triggers PI3K/AKT pathway resulting in apoptosis suppression and chemo resistance induction [143]. BCL2-associated agonist of cell death (Bad) is an apoptotic protein that is phosphorylated and inactivated by AKT thereby it cannot bind and deactivate the BCL-XL [144]. It has been observed that *miR-34c* increased DDP induced cytotoxicity through targeting the MET/PI3K/AKT to reduce Bad phosphorylation in OC cells. Therefore, high levels of dephosphorylated Bad increased apoptosis in ovarian tumor cells [52]. IGF1R is also a tyrosine kinase receptor that promotes cell proliferation and chemo-resistance by triggering the MAPK and PI3K/AKT signaling pathways. *MiR-1294* is also involved in DDP resistance via EMT regulation and IGF1R targeting in OC cells [53]. The mTOR as a member of PI3K family regulates various cellular processes such as cell proliferation, cell migration, and protein synthesis [145]. It also mediates translation of cell cycle regulators such as cyclin A, CDK1/2, and retinoblastoma (Rb) protein. *MiR-199a* increased DDP sensitivity through mTOR targeting in OC cells [54]. It has been reported that *miR-1271* significantly suppressed EMT in DDP-sensitive OC cells via E-cadherin up-regulation and N-cadherin down regulation. *MiR-1271* inhibited the EMT and sensitized OC cells to DDP-treatment via mTOR targeting [55]. The mTOR/p70S6K1 axis has a pivotal role in chemo resistance [146, 147]. A significant *miR-497* down regulation has been observed in DDP-resistant OC cells and tissues. *MiR-497* increased DDP sensitivity via mTOR and p70S6K1 targeting in OC cells [56]. Chemokine receptor 4 (CXCR4) is a receptor of CXC chemokine ligand 12 (CXCL12) involved in tumor progression and drug resistance [148, 149]. EGFR activation up regulates CXCR4 through PI3K/AKT signaling that induces malignant transformation [150]. It has been reported that the ZFAS1 sponged *miR-548e* to up regulate the CXCR4 in OC cells, which induced cell proliferation and DDP resistance. This process was also mediated by *let-7a* down regulation and BCL-XL/S up regulation [57].

**Fig. 3:**
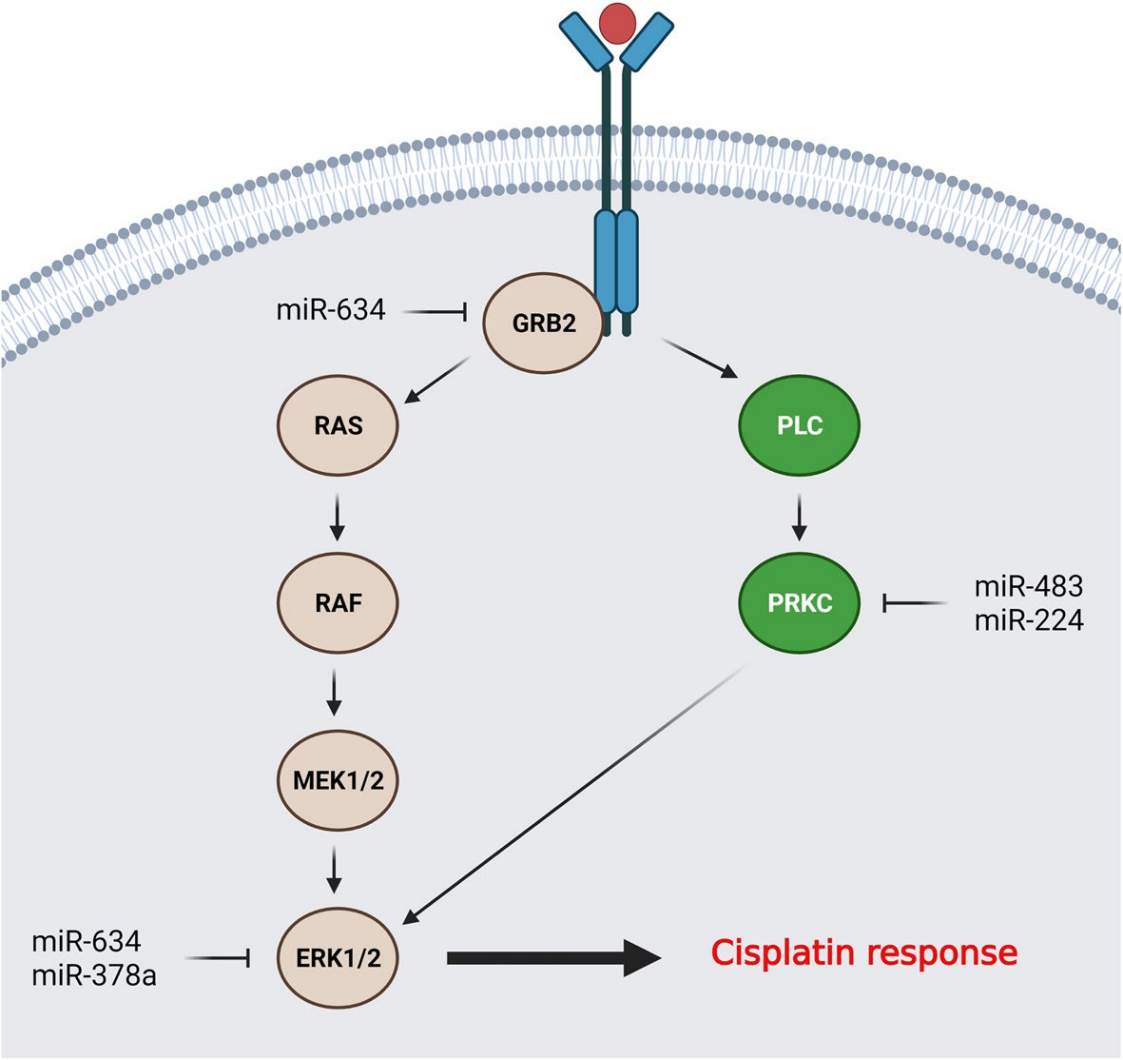
MicroRNAs are involved in regulation of DDP response through MAPK pathway in ovarian tumor cells

MAPK signaling pathway has a critical role in cellular response to cytotoxic agents which is classically activated by receptor tyrosine kinases (RTK). The p38, JNK, and ERK are the main MAPK routs involved in regulation of different cellular processes such as cell cycle, DNA repair, and apoptosis [151–155]. MicroRNAs are involved in regulation of DDP response in ovarian tumor cells through MAPK signaling pathway (Fig. 3). It has been reported that *miR-634* increased DDP sensitivity in OC cells by suppression of G1-S progression and apoptosis induction via CCND1 and MAPK pathway components (GRB2 and ERK2). The MAPK inhibition increased DDP sensitivity which confirmed the miR-634-mediated repression of MAPK pathway as the main molecular mechanism of *miR-634* during DDP resistance in OC cells [58]. LNC00115 up regulation was also observed in OC tissues and DDP-resistant cells. It induced the DDP resistance and cell migration through *miR-7* targeting that resulted in ERK up regulation [59]. *MiR-378a-3p* down-regulations were observed in OC tissues and cell lines. There was also a direct association between the level of *miR-378a-3p* expression and overall survival in OC patients. *MiR-378a-3p* inhibited cell proliferation and sensitized OC cells to DDP via MAPK1/GRB2 suppression [60]. Protein kinase C (PRKC) is activated by diacylglycerol (DAG) or Ca^2+^ that is involved in regulation of cell proliferation, apoptosis, and migration via MAPK signaling activation. It has been reported that there was increased levels of *miR-483-3p* expression in DDP-resistant OC cells that protects them against the DDP mediated DNA damage via PRKCA inhibition [156]. PRKCD as a substrate of CASP3 is also required for apoptosis induction by DDP and doxorubicin [157]. It has been shown that *miR-224-5p* increased DDP resistance by targeting PRKCD [61]. YWHAZ is an adapter protein involved in regulation of different signaling pathways. It has been shown that circ_C20orf11 promoted DDP resistance while reduced apoptosis in DDP-resistant ovarian tumor cell lines through *miR-527* sponging and YWHAZ up regulation [158].

NOTCH and WNT are developmental signaling pathways involved in cell proliferation, differentiation, angiogenesis, apoptosis, and tumor progression [159–162]. Deregulation of JAG1–Notch1 signaling can protect tumor cells toward DDP-mediated apoptosis [163, 164]. It has been shown that there was *miR-449a* down regulation in DDP-resistant OC cells. *MiR-449a* also increased DDP sensitivity by NOTCH1 down regulation and NOTCH signaling inhibition in OC cells [62]. *MiR-338-3p* increased DDP sensitivity through WNT2B inhibition in OC cells. *MiR-338-3p* also repressed EMT process by Vimentin and N-cadherin down regulations and E-cadherin up regulation [63]. Phosphoserine aminotransferase 1 (PSAT1) is involved in serine synthesis that functions as an oncogene during tumor progression and metastasis [165]. It has been reported that *miR-195-5p* inhibited the GSK3β/β-catenin pathway through PSAT1 targeting which reduced angiogenesis and DDP resistance in OC cells. *MiR-195-5p* also down regulated the HIF-1α, VEGF, and β-catenin in OC cells [64].

### Transcription factors and methylation

SOX9 is a developmental transcription factor belonging to the SOX protein family that promotes tumor progression and drug resistance by β-catenin signaling activation [166, 167]. It has been reported that *miR-34c* significantly inhibited OC cell proliferation and DDP-resistance through SOX9 targeting. There was *miR-34c* down regulation, while SOX9, β-catenin, and c-Myc up regulations in OC samples. Moreover, higher levels of *miR-34c* expression was observed in early stage OC patients with longer survival [65]. NEAT1 up regulation was reported in OC cell lines and tissues. It also inhibited DDP-induced apoptosis and increased cell proliferation via *miR-491-5p* sponging and SOX3 up regulation [66]. Runt-related transcription factor 1 (RUNX1) is one of the components of core-binding transcription factors involved in hematopoiesis and leukemia [168]. It has been observed that the RUNX1 up regulation reduced the overall survival of OC patients. RUNX1 also decreased DDP-induced apoptosis by BCL2 regulation via miR-17 ~ 92 cluster in OC [169]. Forkhead box gene P1 (FOXP1) transcription factor has a pivotal role during embryogenesis and heart development in humans [170]. Beclin1, LC3, and P62 are involved in autophagy process in mammals [171–173]. It has been shown that increased ratio of LC3-II/LC3-I, up regulated the Beclin1 and MDR-1, and down regulated the P62 in DDP-resistant OC cells. Moreover, *miR-29c-3p* regulated DDP resistance through FOXP1 inhibition in OC cells [174]. C-MYB oncogene belongs to the myeloblastosis (MYB) transcription factors associated with DDP resistance in OC [175]. It has been shown that there was a direct correlation between c-MYB and *miR-21* expression levels. EMT process and DDP resistance were also induced following the c-MYB up regulation. C-MYB activated the WNT signaling via reduction of β-catenin phosphorylation. The ES2 cell lines with c-MYB and *miR-21* over expressions were more aggressive and DDP resistant compared with controls [67]. C-JUN is an oncogenic transcription factor that induces cell proliferation and migration [176]. It has been reported that there was significant *miR-139-5p* down regulation in DDP-resistant OC cells in comparison with parental cells. *MiR-139-5p* also reversed DDP resistance via C-JUN targeting. Moreover, *miR-139-5p* inhibited the c-JUN and ATF2 interaction that resulted in BCL-XL down regulation and DDP-mediated apoptosis in OC cells [68]. Urothelial carcinoma associated 1 (UCA1) is an lncRNA that affects the tumor progression via regulation of WNT pathway [177]. FOSL2 belongs to the FOS family of transcription factors that binds with JUN to form the AP-1 transcriptional complex involving in regulation of cell proliferation and differentiation. It was observed that there were UCA1 up regulations in DDP-resistant OC cells and tissues. UCA1 promoted DDP resistance by *miR-143* sponging which resulted in FOSL2 down regulation [69]. STAT3 is a transcription factor that is phosphorylated and activated by interferons, interleukins, and growth factors for the nuclear translocation. It is involved in tumor progression via regulation of various oncogenes such as c-MYC and CCND1 [178]. It has been shown that STAT3 up regulated *miR-216a* that increased DDP resistance in OC cells [179]. E2F3 is a key transcription factor involved in cell cycle regulation [180]. E2F3a over expression has been associated with tumor stage in OC patients [181]. It has been reported that there was a significant *miR-210-3p* down regulation in DDP-resistant compared with sensitive OC cells. *Mir-210-3p* increased DDP-response through E2F3 targeting [70]. C-MYC is also an oncogenic transcription factor that regulates cell proliferation [182]. It is amplified in 30–60% of ovarian tumors and is associated with drug-resistance [183, 184]. It has been reported that there was significant *miR-137* down regulation in DDP resistant OC cells. C-MYC inhibited the *miR-137* expression via EZH2 recruitment. Increased levels of ROS induced c-MYC expression which up regulated the EZH2 through *miR-137* inhibition [71].

Epithelial mesenchymal transition (EMT) is considered one of the key mechanisms of chemo resistance [185]. EMT process is orchestrated by a molecular signature including E-cadherin down regulation, while vimentin, N-cadherin, Fibronectin, and Snail, TWIST, ZEB1, and ZEB2 up regulations. EMT-specific transcription factors are also correlated with chemo resistance [186]. TWIST1 as a bHLH transcription factor is the main inducer of EMT via E-cadherin down regulation by the BMI-1 chromatin remodeling factor [187]. It has been reported that there were higher levels of TWIST1 and vimentin expressions in DDP-resistant compared with DDP-sensitive ovarian tumors which was correlated with a lower relapse time and poor prognosis among EOC patients. *MiR-186* down regulated the TWIST1 and EMT-associated markers that resulted in EMT alteration and DDP sensitivity in EOC [72]. A significant *miR-363* down regulation has been shown in malignant EOC in comparison with benign tissues which was associated with high FIGO stage and chemo resistance. *MiR-363* also reduced DDP-resistance through SNAIL targeting in EOC cells [73]. BMI-1 is a proto-oncogene involved in regulation of cell proliferation and cancer stem cells (CSCs) maintenance. It has been observed that the SKOV3/DDP cell line had significantly lower levels of *miR-132* expression compared with parental SKOV3 cell line. *MiR-132* down regulation induced DDP resistance of in OC via BMI-1 targeting and apoptosis inhibition [74]. There was a significant HOTTIP up regulation in DDP resistant ovarian tumor cells. HOTTIP increased DDP resistance in ovarian tumor cells by *miR-205* sponging and ZEB2 up regulation [75].

EZH2 is one of the components of PRC2 complex that is involved in regulation of cell proliferation, chemo resistance, and embryogenesis via catalyzing the histone 3 lysine 27 methylation [188–190]. *MiR-101* down regulation has been observed in EOC tissues. There was also a significant inverse association between the levels of *miR-101* expression, grade, and FIGO stage. Moreover, *miR-101* reduced OC cell proliferation and migration and increased DDP-induced cytotoxicity by EZH2 targeting [76]. HOX transcript antisense RNA (HOTAIR) is an lncRNA that has been frequently reported to be involved in tumor progression [191]. There was an inverse association between HOTAIR and *miR-138-5p* in SKOV3/DDP and A2780/DDP cells in which HOTAIR down regulation and *miR-138-5p* up regulation increased DDP sensitivity. *MiR-138-5p* also regulated the EZH2 and SIRT1 expressions that could be associated with DDP resistance in OC cells [77]. RNF2 belongs to the PRC family of proteins that is regulated by MAPK signaling pathway [192]. It has been observed that there was *miR-139-5p* down regulation in DDP-resistant OC tissues. *MiR-139-5p* induced DDP mediated apoptosis through RNF2 inhibition and MAPK signaling inactivation in OC cells [78].

Hypoxia is commonly caused due to rapid tumor cell proliferation in tumor microenvironment which is associated with chemo resistance. Hypoxia-inducible factor 1 (Hif1) is a pivotal transcription factor involved in hypoxia response through DNA repair induction and apoptosis inhibition [193]. It has been reported that there was significant reduced levels of *miR-199a* expressions in ovarian tumors compared with normal margins. *MiR-199a* was also down regulated in DDP-resistant in comparison with sensitive OC cells. *MiR-199a* increased DDP sensitivity through Hif1α targeting in OC cells [79].

Epigenetic modifications by DNA hypermethylation can be involved in DDP resistance of OC cells [194]. DNA methylation at cytosine residues is a pivotal mechanism of epigenetic regulation that can be done by DNMT1 as a critical enzyme for hemimethylated DNA preservation during DNA replication and silencing of tumor suppressors [195–197]. It has been reported that there was a significant *miR-30a/c-5p* down regulation in DDP-resistant OC cells compared with sensitive controls. *MiR-30a/c-5p* reduced the levels of DNMT1 and Snail, whereas DNMT1 also down regulated the *miR-30a/c-5p* through promoter methylation. DNMT1 mediated down regulation of *miR-30a/c-5p* increased DDP resistance and EMT through elimination of inhibitory role of *miR-30a/c-5p* on DNMT1 and Snail in OC cells [80]. Another study also showed significant *miR-152* and *miR-185* down regulations in DDP-resistant OC cells compared with sensitive cells. These microRNAs increased DDP sensitivity by DNMT1 targeting in OC [81]. *MiR-200b/c* also increased DDP sensitivity via direct DNMT3A/DNMT3B targeting and indirect DNMT1 down regulation by Sp1 targeting in ovarian tumor cells [82].

### Transporters and structural factors

ATP-binding cassette (ABC) transporters are well studied causes of chemo resistance which transport various compounds and substrates such as nutrients, lipids, and drugs across membranes. ABC transporters are involved in drug response of ovarian tumor cells via increasing the efflux of chemotherapeutic drugs [198]. ABCB1 belongs to the ABC transporter family [199]. The GST-π isoform as a member of the GST family is involved in detoxification of cytostatic agents which influences the efficiency of chemo therapeutic treatment and patients’ survival [200]. It has been reported that *miR-186* increased the DDP sensitivity of OC cells via ABCB1 and GST-π down regulations [83]. *MiR-595* down regulation has been observed in OC tissues and cell lines. There were also lower levels of *miR-595* expression in metastatic lymph nodes compared with OC tissues and normal margins. Moreover, *miR-595* inhibited the OC cell proliferation, metastasis, and DDP resistance via ABCB1 suppression [84]. It has been observed that *miR-130a* up regulation can be correlated with DDP resistance via ABCB1 regulation in OC cells [85]. *MiR-873* also increased the DDP and paclitaxel sensitivity through ABCB1 targeting in OC cells [86]. Another study has been shown that *miR-514* inhibited ovarian tumor cell proliferation and increased DDP sensitivity via ABCA1, ABCA10, and ABCF2 targeting [87].

SLC40A1 is involved in intracellular iron trans-membrane flow [201]. SLC40A1 can be inhibited by NRF2 during DDP sensitization in OC [202]. It has been reported that *miR-194-5p* induced DDP resistance through SLC40A1 targeting [88]. Cellular copper homeostasis is maintained by copper-transporting ATPases [203]. They trapped platinum compounds inside vesicular structures to prevent their cellular effect [204]. It has been observed that there was *miR-139* down-regulation in DDP-resistant OC tissues and cell lines. *MiR-139* sensitized OC cells toward DDP via ATP7A/B targeting [89].

Extra cellular matrix (ECM) can also be involved in DDP resistance in OC via manipulation of survival signal transduction due DDP treatment. It has been reported that miR-29 knockdown increased DDP resistance by COL1A1 up regulation [90]. ITGB8 belongs to the integrin β-chain family that is involved in cell growth, migration, and drug resistance [205, 206]. It has been reported that there was significant *miR-199a-3p* down regulation in DDP-resistant OC tissues and cell lines. *MiR-199a-3p* increased DDP sensitivity through ITGB8 suppression in OC cells [91]. Syndecan 4 (SDC4) is a trans-membrane proteoglycan that has pivotal role in regulation of intracellular signaling pathways as a receptor. It has been observed that WDFY3-AS2 silencing significantly suppressed A2780-DDP cell proliferation while promoted apoptosis. WDFY3-AS2 was involved in SDC4 up regulation through *miR-139-5p* sponging [92]. TRPM2-AS also induced DDP resistance through *miR-138-5p* sponging and SDC3 up regulation in OC cells [93]. Contactin 1 (CNTN1) belongs to the immunoglobulin superfamily that functions in cell adhesion. It has pivotal roles in axon connections and nervous system development. LINC00184 significantly promoted DDP resistance via *miR-1305* sponging that resulted in CNTN1 up regulation in OC cells [94].

KCNMA1 is a pore-forming component of BK channels expressed by many cell types that is involved in variety of stimuli and tumor progression [207, 208]. It has been observed that *miR-31* increased DDP resistance in OC cells via KCNMA1 suppression [95]. NF-E2-related factor 2 (NRF2) is a regulator of cytoprotective factors that has normally a low level of expression, while it is dramatically up regulated upon chemical or reactive oxygen species (ROS) exposures. KEAP1 is a component of E3 ubiquitin ligase complex that is associated with rapid NRF2 degradation in normal condition [209]. *MiR-141* up regulation has been observed in DDP resistant OC cells. It induced NF-kB signaling and down regulated the KEAP1 [96].

## Conclusions

Although, DDP is one of the common chemotherapeutic compounds used for OC treatment; there is a high ratio of DDP resistance among OC patients. Since, DDP has severe side effects; it is required to clarify the molecular mechanisms involved in DDP resistance to find novel efficient therapeutic modalities to improve the clinical outcomes of OC patients. MiRNAs are non-invasive and more stable factors compared with mRNAs. It was observed that miRNAs exert their role on DDP response mainly through regulation of apoptosis, signaling pathways, and transcription factors in OC cells. This review highlighted the miRNAs as important regulators of DDP response in ovarian tumor cells. Moreover, present review paves the way of suggesting a non-invasive panel of prediction markers for DDP response among OC patients. Suppression or replacement therapy can be used regarding the up regulation or down regulation of miRNAs in ovarian tumor cells, respectively. MiRNAs can also be used as prognostic markers in ovarian cancer patients. It seems that miRNAs have higher therapeutic efficiency compared with siRNA. However, majority of the studies are in the level of in vitro studies. Therefore, based on the complex in vivo environment, miRNAs may exhibit different molecular behaviors in vitro compared with in vivo which highlights the priority of the in vivo studies before the introduction of prognostic miRNA-based panel markers.

## Data Availability

The datasets used and/or analyzed during the current study are available from the corresponding author on reasonable request.

## Abbreviations

OC: Ovarian cancer
DDP: Platinum diamminodichloride
miRNAs: MicroRNAs
IAPs: Inhibitors of apoptosis proteins
LncRNAs: Long non coding RNAs
ceRNA: Competing endogenous RNA
CCAT1: Colon cancer-associated transcript 1
NF1: Neurofibromatosis type 1
LGALS3: Galectin-3
NER: Nucleotide excision repair
CAFs: Cancer-associated fibroblasts
APC: Anaphase-promoting complex
PTEN: Phosphatase and tensin homolog
Bad: BCL2-associated agonist of cell death
Rb: Retinoblastoma
CXCR4: Chemokine receptor 4
CXCL12: CXC chemokine ligand 12
PRKC: Protein kinase C
DAG: Diacylglycerol
PSAT1: Phosphoserine aminotransferase 1
RUNX1: Runt-related transcription factor 1
FOXP1: Forkhead box gene P1
UCA1: Urothelial carcinoma associated 1
EMT: Epithelial mesenchymal transition
CSCs: Cancer stem cells
HOTAIR: HOX transcript antisense RNA
Hif1: Hypoxia-inducible factor 1
ABC: ATP-binding cassette
ECM: Extra cellular matrix
NRF2: NF-E2-related factor 2
ROS: Reactive oxygen species
MYB: Myeloblastosis
SDC4: Syndecan 4
CNTN1: Contactin 1

## References

[1] Siegel R, Naishadham D, Jemal A (2013). Cancer statistics, 2013. CA Cancer J Clin.

[2] Al-Alem L (2011). Specific thiazolidinediones inhibit ovarian cancer cell line proliferation and cause cell cycle arrest in a PPARgamma independent manner. PLoS One.

[3] Yang HP (2012). Ovarian cancer risk factors by histologic subtypes in the NIH-AARP diet and health study. Int J Cancer.

[4] Mortazavi H (2020). Potential cytotoxic and anti-metastatic effects of berberine on gynaecological cancers with drug-associated resistance. Eur J Med Chem.

[5] Langhe R (2015). microRNA and ovarian cancer. Adv Exp Med Biol.

[6] Jessmon P (2017). Epidemiology and treatment patterns of epithelial ovarian cancer. Expert Rev Anticancer Ther.

[7] Lokadasan R (2016). Targeted agents in epithelial ovarian cancer: review on emerging therapies and future developments. Ecancermedicalscience.

[8] Muggia F (2009). Platinum compounds 30 years after the introduction of cisplatin: implications for the treatment of ovarian cancer. Gynecol Oncol.

[9] Vaughan S (2011). Rethinking ovarian cancer: recommendations for improving outcomes. Nat Rev Cancer.

[10] Reed NS, Sadozye AH (2005). Role of chemotherapy in the management of epithelial ovarian cancer. Expert Rev Anticancer Ther.

[11] Li J (2016). Overexpression of long non-coding RNA HOTAIR leads to chemoresistance by activating the Wnt/beta-catenin pathway in human ovarian cancer. Tumour Biol.

[12] Bonanno L, Favaretto A, Rosell R (2014). Platinum drugs and DNA repair mechanisms in lung cancer. Anticancer Res.

[13] Kigawa J (2013). New strategy for overcoming resistance to chemotherapy of ovarian cancer. Yonago Acta Med.

[14] Sui X (2013). Autophagy and chemotherapy resistance: a promising therapeutic target for cancer treatment. Cell Death Dis.

[15] Galluzzi L (2012). Molecular mechanisms of cisplatin resistance. Oncogene.

[16] Siddik ZH (2003). Cisplatin: mode of cytotoxic action and molecular basis of resistance. Oncogene.

[17] Garzon R, Croce CM (2011). MicroRNAs and cancer: introduction. Semin Oncol.

[18] Mendell JT (2005). MicroRNAs: critical regulators of development, cellular physiology and malignancy. Cell Cycle.

[19] Boren T (2009). MicroRNAs and their target messenger RNAs associated with ovarian cancer response to chemotherapy. Gynecol Oncol.

[20] Merritt WM (2008). Dicer, Drosha, and outcomes in patients with ovarian cancer. N Engl J Med.

[21] Wang Y (2018). miR-98-5p contributes to cisplatin resistance in epithelial ovarian cancer by suppressing miR-152 biogenesis via targeting Dicer1. Cell Death Dis.

[22] Chen W (2017). miR-509-3p promotes cisplatin-induced apoptosis in ovarian cancer cells through the regulation of anti-apoptotic genes. Pharmacogenomics.

[23] Li X (2019). miR-142-5p enhances cisplatin-induced apoptosis in ovarian cancer cells by targeting multiple anti-apoptotic genes. Biochem Pharmacol.

[24] Li X (2017). MicroRNA146a5p enhances cisplatininduced apoptosis in ovarian cancer cells by targeting multiple antiapoptotic genes. Int J Oncol.

[25] Chen W (2016). MicroRNA-509-3p increases the sensitivity of epithelial ovarian cancer cells to cisplatin-induced apoptosis. Pharmacogenomics.

[26] Pang Y (2014). MiR-519d represses ovarian cancer cell proliferation and enhances cisplatin-mediated cytotoxicity in vitro by targeting XIAP. Onco Targets Ther.

[27] Zhang X (2013). Downregulation of miR-130a contributes to cisplatin resistance in ovarian cancer cells by targeting X-linked inhibitor of apoptosis (XIAP) directly. Acta Biochim Biophys Sin Shanghai.

[28] Chen W (2016). MicroRNA-155 promotes apoptosis in SKOV3, A2780, and primary cultured ovarian cancer cells. Tumour Biol.

[29] Liu R, Guo H, Lu S (2018). MiR-335-5p restores cisplatin sensitivity in ovarian cancer cells through targeting BCL2L2. Cancer Med.

[30] Wang DY, Li N, Cui YL (2020). Long non-coding RNA CCAT1 sponges miR-454 to promote Chemoresistance of ovarian cancer cells to Cisplatin by regulation of surviving. Cancer Res Treat.

[31] Rao YM (2013). MiR-106a targets mcl-1 to suppress cisplatin resistance of ovarian cancer A2780 cells. J Huazhong Univ Sci Technolog Med Sci.

[32] Chen W (2020). microRNA-137 downregulates MCL1 in ovarian cancer cells and mediates cisplatin-induced apoptosis. Pharmacogenomics.

[33] Su J (2019). NF1 regulates apoptosis in ovarian cancer cells by targeting MCL1 via miR-142-5p. Pharmacogenomics.

[34] Zuo Y (2020). MiR-34a-5p/PD-L1 axis regulates cisplatin chemoresistance of ovarian cancer cells. Neoplasma.

[35] Bieg D (2019). MiR-424-3p suppresses galectin-3 expression and sensitizes ovarian cancer cells to cisplatin. Arch Gynecol Obstet.

[36] Kong F (2011). miR-125b confers resistance of ovarian cancer cells to cisplatin by targeting pro-apoptotic Bcl-2 antagonist killer 1. J Huazhong Univ Sci Technolog Med Sci.

[37] Echevarria-Vargas IM, Valiyeva F, Vivas-Mejia PE (2014). Upregulation of miR-21 in cisplatin resistant ovarian cancer via JNK-1/c-Jun pathway. PLoS One.

[38] Li H (2014). microRNA-106a modulates cisplatin sensitivity by targeting PDCD4 in human ovarian cancer cells. Oncol Lett.

[39] Wambecke A, et al. The lncRNA 'UCA1' modulates the response to chemotherapy of ovarian cancer through direct binding to miR-27a-5p and control of UBE2N levels. Mol Oncol. 2021. 10.1002/1878-0261.13045 PMC8637575 34160887

[40] Liu Y (2017). MiR-216b increases cisplatin sensitivity in ovarian cancer cells by targeting PARP1. Cancer Gene Ther.

[41] Zhu M, Yang L, Wang X (2020). NEAT1 knockdown suppresses the Cisplatin resistance in ovarian cancer by regulating miR-770-5p/PARP1 Axis. Cancer Manag Res.

[42] Sun C (2013). miR-9 regulation of BRCA1 and ovarian cancer sensitivity to cisplatin and PARP inhibition. J Natl Cancer Inst.

[43] Zhao H (2016). MiR-770-5p inhibits cisplatin chemoresistance in human ovarian cancer by targeting ERCC2. Oncotarget.

[44] Guo H (2019). Cancer-associated fibroblast-derived exosomal microRNA-98-5p promotes cisplatin resistance in ovarian cancer by targeting CDKN1A. Cancer Cell Int.

[45] Guo P (2016). miR-100 resensitizes resistant epithelial ovarian cancer to cisplatin. Oncol Rep.

[46] Cheng Y, et al. MiRNA-409-3p enhances cisplatin-sensitivity of ovarian cancer cells by blocking the autophagy mediated by Fip200. Oncol Res. 2018. 10.3727/096504017X15138991620238 29295727

[47] Wu D (2018). Downregulation of miR-503 contributes to the development of drug resistance in ovarian cancer by targeting PI3K p85. Arch Gynecol Obstet.

[48] Qin X, Sun L, Wang J (2017). Restoration of microRNA-708 sensitizes ovarian cancer cells to cisplatin via IGF2BP1/Akt pathway. Cell Biol Int.

[49] Shi X (2018). miR-205-5p mediated Downregulation of PTEN contributes to Cisplatin resistance in C13K human ovarian cancer cells. Front Genet.

[50] Fu X (2012). Involvement of microRNA-93, a new regulator of PTEN/Akt signaling pathway, in regulation of chemotherapeutic drug cisplatin chemosensitivity in ovarian cancer cells. FEBS Lett.

[51] Li L, et al. Long noncoding RNA HAND2AS1/miR106a/PTEN axis resensitizes cisplatinresistant ovarian cells to cisplatin treatment. Mol Med Rep. 2021;24(5):1–10. 10.3892/mmr.2021.12402 PMC8436234 34476500

[52] Yang S, Li Z, Luo R (2020). miR-34c targets MET to improve the anti-tumor effect of Cisplatin on ovarian cancer. Onco Targets Ther.

[53] Zhang Y (2018). MiR-1294 confers cisplatin resistance in ovarian cancer cells by targeting IGF1R. Biomed Pharmacother.

[54] Wang Z (2013). microRNA-199a is able to reverse cisplatin resistance in human ovarian cancer cells through the inhibition of mammalian target of rapamycin. Oncol Lett.

[55] Chen Y, Wang L, Zhou J (2019). Effects of microRNA-1271 on ovarian cancer via inhibition of epithelial-mesenchymal transition and cisplatin resistance. J Obstet Gynaecol Res.

[56] Xu S (2015). MiR-497 decreases cisplatin resistance in ovarian cancer cells by targeting mTOR/P70S6K1. Oncotarget.

[57] Zhang J (2020). miR-548e sponged by ZFAS1 regulates metastasis and Cisplatin resistance of OC by targeting CXCR4 and let-7a/BCL-XL/S signaling Axis. Mol Ther Nucleic Acids.

[58] van Jaarsveld MT (2015). miR-634 restores drug sensitivity in resistant ovarian cancer cells by targeting the Ras-MAPK pathway. Mol Cancer.

[59] Jiang X (2021). LNC00115 mediates Cisplatin resistance by regulating the miR-7/ERK Signalling pathway in ovarian cancer. Cancer Manag Res.

[60] Xu ZH, Yao TZ, Liu W (2018). miR-378a-3p sensitizes ovarian cancer cells to cisplatin through targeting MAPK1/GRB2. Biomed Pharmacother.

[61] Zhao H (2014). Expression of miR-224-5p is associated with the original cisplatin resistance of ovarian papillary serous carcinoma. Oncol Rep.

[62] Zhou Y (2014). MicroRNA-449a reduces cell survival and enhances cisplatin-induced cytotoxicity via downregulation of NOTCH1 in ovarian cancer cells. Tumour Biol.

[63] Niu Q (2019). MiR-338-3p enhances ovarian cancer cell sensitivity to Cisplatin by Downregulating WNT2B. Yonsei Med J.

[64] Dai J (2019). Overexpression of microRNA-195-5p reduces cisplatin resistance and angiogenesis in ovarian cancer by inhibiting the PSAT1-dependent GSK3beta/beta-catenin signaling pathway. J Transl Med.

[65] Xiao S (2019). MiR-34c/SOX9 axis regulates the chemoresistance of ovarian cancer cell to cisplatin-based chemotherapy. J Cell Biochem.

[66] Jia X, Wei L, Zhang Z (2021). NEAT1 overexpression indicates a poor prognosis and induces chemotherapy resistance via the miR-491-5p/SOX3 signaling pathway in ovarian cancer. Front Genet.

[67] Zhang XY (2020). Regulation of MYB mediated cisplatin resistance of ovarian cancer cells involves miR-21-wnt signaling axis. Sci Rep.

[68] Jiang Y (2018). Recovery of miR-139-5p in ovarian cancer reverses Cisplatin resistance by targeting C-Jun. Cell Physiol Biochem.

[69] Li Z (2019). lncRNA UCA1 mediates resistance to Cisplatin by regulating the miR-143/FOSL2-signaling pathway in ovarian cancer. Mol Ther Nucleic Acids.

[70] Jin Y (2019). miR2103p regulates cell growth and affects cisplatin sensitivity in human ovarian cancer cells via targeting E2F3. Mol Med Rep.

[71] Sun J (2019). miR-137 mediates the functional link between c-Myc and EZH2 that regulates cisplatin resistance in ovarian cancer. Oncogene.

[72] Zhu X (2016). miR-186 regulation of Twist1 and ovarian cancer sensitivity to cisplatin. Oncogene.

[73] Cao L (2018). MiR-363 inhibits cisplatin chemoresistance of epithelial ovarian cancer by regulating snail-induced epithelial-mesenchymal transition. BMB Rep.

[74] Zhang XL (2019). MicroRNA-132 reverses cisplatin resistance and metastasis in ovarian cancer by the targeted regulation on Bmi-1. Eur Rev Med Pharmacol Sci.

[75] Dong YJ, Feng W, Li Y (2021). HOTTIP-miR-205-ZEB2 Axis confers Cisplatin resistance to ovarian cancer cells. Front Cell Dev Biol.

[76] Liu L (2014). miR-101 regulates expression of EZH2 and contributes to progression of and cisplatin resistance in epithelial ovarian cancer. Tumour Biol.

[77] Zhang Y (2020). Knockdown of long non-coding RNA HOTAIR reverses cisplatin resistance of ovarian cancer cells through inhibiting miR-138-5p-regulated EZH2 and SIRT1. Biol Res.

[78] Chen Y (2018). Reversal of cisplatin resistance by microRNA-139-5p-independent RNF2 downregulation and MAPK inhibition in ovarian cancer. Am J Phys Cell Phys.

[79] Feng X (2017). miR-199a modulates cisplatin resistance in ovarian cancer by targeting Hif1alpha. Onco Targets Ther.

[80] Han X (2017). A feedback loop between miR-30a/c-5p and DNMT1 mediates Cisplatin resistance in ovarian cancer cells. Cell Physiol Biochem.

[81] Xiang Y (2014). MiR-152 and miR-185 co-contribute to ovarian cancer cells cisplatin sensitivity by targeting DNMT1 directly: a novel epigenetic therapy independent of decitabine. Oncogene.

[82] Liu J (2019). miR-200b and miR-200c co-contribute to the cisplatin sensitivity of ovarian cancer cells by targeting DNA methyltransferases. Oncol Lett.

[83] Sun KX (2015). MicroRNA-186 induces sensitivity of ovarian cancer cells to paclitaxel and cisplatin by targeting ABCB1. J Ovarian Res.

[84] Tian S (2016). MicroRNA-595 sensitizes ovarian cancer cells to cisplatin by targeting ABCB1. Oncotarget.

[85] Yang L (2012). Altered microRNA expression in cisplatin-resistant ovarian cancer cells and upregulation of miR-130a associated with MDR1/P-glycoprotein-mediated drug resistance. Oncol Rep.

[86] Wu DD (2016). MicroRNA-873 mediates multidrug resistance in ovarian cancer cells by targeting ABCB1. Tumour Biol.

[87] Xiao S (2018). MiR-514 attenuates proliferation and increases chemoresistance by targeting ATP binding cassette subfamily in ovarian cancer. Mol Gen Genomics.

[88] Wu J (2020). miR-194-5p inhibits SLC40A1 expression to induce cisplatin resistance in ovarian cancer. Pathol Res Pract.

[89] Xiao F (2018). MircroRNA-139 sensitizes ovarian cancer cell to cisplatin-based chemotherapy through regulation of ATP7A/B. Cancer Chemother Pharmacol.

[90] Yu PN (2014). Downregulation of miR-29 contributes to cisplatin resistance of ovarian cancer cells. Int J Cancer.

[91] Cui Y (2018). miR-199a-3p enhances cisplatin sensitivity of ovarian cancer cells by targeting ITGB8. Oncol Rep.

[92] Wu Y (2021). LncRNA WDFY3-AS2 promotes cisplatin resistance and the cancer stem cell in ovarian cancer by regulating hsa-miR-139-5p/SDC4 axis. Cancer Cell Int.

[93] Ding Y (2021). LncRNA TRPM2-AS promotes ovarian cancer progression and cisplatin resistance by sponging miR-138-5p to release SDC3 mRNA. Aging (Albany NY).

[94] Han Y (2021). LINC00184 promotes ovarian cancer cells proliferation and Cisplatin resistance by elevating CNTN1 expression via sponging miR-1305. Onco Targets Ther.

[95] Samuel P (2016). Over-expression of miR-31 or loss of KCNMA1 leads to increased cisplatin resistance in ovarian cancer cells. Tumour Biol.

[96] van Jaarsveld MT (2013). miR-141 regulates KEAP1 and modulates cisplatin sensitivity in ovarian cancer cells. Oncogene.

[97] Berthelet J, Dubrez L (2013). Regulation of apoptosis by inhibitors of apoptosis (IAPs). Cells.

[98] Alhourani E (2016). BIRC3 alterations in chronic and B-cell acute lymphocytic leukemia patients. Oncol Lett.

[99] Wagenknecht B (1999). Expression and biological activity of X-linked inhibitor of apoptosis (XIAP) in human malignant glioma. Cell Death Differ.

[100] Adams JM, Cory S (2007). The Bcl-2 apoptotic switch in cancer development and therapy. Oncogene.

[101] Cory S, Huang DC, Adams JM (2003). The Bcl-2 family: roles in cell survival and oncogenesis. Oncogene.

[102] Frenzel A (2009). Bcl2 family proteins in carcinogenesis and the treatment of cancer. Apoptosis.

[103] Nissan A (2012). Colon cancer associated transcript-1: a novel RNA expressed in malignant and pre-malignant human tissues. Int J Cancer.

[104] Simonin K (2009). Mcl-1 is an important determinant of the apoptotic response to the BH3-mimetic molecule HA14-1 in cisplatin-resistant ovarian carcinoma cells. Mol Cancer Ther.

[105] Yuan Z (2011). The p53 upregulated modulator of apoptosis (PUMA) chemosensitizes intrinsically resistant ovarian cancer cells to cisplatin by lowering the threshold set by Bcl-x(L) and mcl-1. Mol Med.

[106] Bollag G (1996). Loss of NF1 results in activation of the Ras signaling pathway and leads to aberrant growth in haematopoietic cells. Nat Genet.

[107] Cox AD, Der CJ (2010). Ras history: the saga continues. Small GTPases.

[108] Dasgupta B (2005). Proteomic analysis reveals hyperactivation of the mammalian target of rapamycin pathway in neurofibromatosis 1-associated human and mouse brain tumors. Cancer Res.

[109] Arima Y (2010). Decreased expression of neurofibromin contributes to epithelial-mesenchymal transition in neurofibromatosis type 1. Exp Dermatol.

[110] Holzel M (2010). NF1 is a tumor suppressor in neuroblastoma that determines retinoic acid response and disease outcome. Cell.

[111] Smith HJ (2017). Epigenetic therapy for the treatment of epithelial ovarian cancer: a clinical review. Gynecol Oncol Rep.

[112] Shalapour S (2015). Immunosuppressive plasma cells impede T-cell-dependent immunogenic chemotherapy. Nature.

[113] Szczepanski MJ (2009). Increased frequency and suppression by regulatory T cells in patients with acute myelogenous leukemia. Clin Cancer Res.

[114] Cho H, et al. Programmed cell death 1 (PD-1) and cytotoxic T lymphocyte-associated antigen 4 (CTLA-4) in viral hepatitis. Int J Mol Sci. 2017;18(7):1517. 10.3390/ijms18071517 PMC5536007 28703774

[115] Keir ME, Francisco LM, Sharpe AH (2007). PD-1 and its ligands in T-cell immunity. Curr Opin Immunol.

[116] Mandai M (2016). Anti-PD-L1/PD-1 immune therapies in ovarian cancer: basic mechanism and future clinical application. Int J Clin Oncol.

[117] Sheng Q (2020). Cisplatin-mediated down-regulation of miR-145 contributes to up-regulation of PD-L1 via the c-Myc transcription factor in cisplatin-resistant ovarian carcinoma cells. Clin Exp Immunol.

[118] Kobayashi T (2011). Transient silencing of galectin-3 expression promotes both in vitro and in vivo drug-induced apoptosis of human pancreatic carcinoma cells. Clin Exp Metastasis.

[119] Pokrywka M (2016). Gal-3 does not suppress cisplatin-induced apoptosis in A-375 melanoma cells. Cell Biol Int.

[120] Zhang H (2006). Involvement of programmed cell death 4 in transforming growth factor-beta1-induced apoptosis in human hepatocellular carcinoma. Oncogene.

[121] Sakthivel KM, Hariharan S (2017). Regulatory players of DNA damage repair mechanisms: role in cancer Chemoresistance. Biomed Pharmacother.

[122] Beck C (2014). Poly(ADP-ribose) polymerases in double-strand break repair: focus on PARP1, PARP2 and PARP3. Exp Cell Res.

[123] Reynolds P (2015). Disruption of PARP1 function inhibits base excision repair of a sub-set of DNA lesions. Nucleic Acids Res.

[124] Hu Y (2014). PARP1-driven poly-ADP-ribosylation regulates BRCA1 function in homologous recombination-mediated DNA repair. Cancer Discov.

[125] Bhattacharyya A (2000). The breast cancer susceptibility gene BRCA1 is required for subnuclear assembly of Rad51 and survival following treatment with the DNA cross-linking agent cisplatin. J Biol Chem.

[126] Wang B (2007). Abraxas and RAP80 form a BRCA1 protein complex required for the DNA damage response. Science.

[127] Kartalou M, Essigmann JM (2001). Recognition of cisplatin adducts by cellular proteins. Mutat Res.

[128] Kim SH (2012). Clinical significance of ERCC2 haplotype-tagging single nucleotide polymorphisms in patients with unresectable non-small cell lung cancer treated with first-line platinum-based chemotherapy. Lung Cancer.

[129] Vafaee F (2017). Functional prediction of long non-coding RNAs in ovarian cancer-associated fibroblasts indicate a potential role in metastasis. Sci Rep.

[130] Azmi AS, Bao B, Sarkar FH (2013). Exosomes in cancer development, metastasis, and drug resistance: a comprehensive review. Cancer Metastasis Rev.

[131] Stivala LA, Cazzalini O, Prosperi E (2012). The cyclin-dependent kinase inhibitor p21CDKN1A as a target of anti-cancer drugs. Curr Cancer Drug Targets.

[132] Degenhardt Y, Lampkin T (2010). Targeting polo-like kinase in cancer therapy. Clin Cancer Res.

[133] Ersahin T, Tuncbag N, Cetin-Atalay R (2015). The PI3K/AKT/mTOR interactive pathway. Mol BioSyst.

[134] Gohr K (2017). Inhibition of PI3K/Akt/mTOR overcomes cisplatin resistance in the triple negative breast cancer cell line HCC38. BMC Cancer.

[135] Dobbin ZC, Landen CN (2013). The importance of the PI3K/AKT/MTOR pathway in the progression of ovarian cancer. Int J Mol Sci.

[136] Tsuruo T (2003). Molecular targeting therapy of cancer: drug resistance, apoptosis and survival signal. Cancer Sci.

[137] Gottlieb TM (2002). Cross-talk between Akt, p53 and Mdm2: possible implications for the regulation of apoptosis. Oncogene.

[138] Hayakawa J (2000). Inhibition of BAD phosphorylation either at serine 112 via extracellular signal-regulated protein kinase cascade or at serine 136 via Akt cascade sensitizes human ovarian cancer cells to cisplatin. Cancer Res.

[139] Bell JL (2013). Insulin-like growth factor 2 mRNA-binding proteins (IGF2BPs): post-transcriptional drivers of cancer progression?. Cell Mol Life Sci.

[140] Li H, Zeng J, Shen K (2014). PI3K/AKT/mTOR signaling pathway as a therapeutic target for ovarian cancer. Arch Gynecol Obstet.

[141] Xiang Y (2020). MiR-186 bidirectionally regulates cisplatin sensitivity of ovarian cancer cells via suppressing targets PIK3R3 and PTEN and upregulating APAF1 expression. J Cancer.

[142] Ho-Yen CM, Jones JL, Kermorgant S (2015). The clinical and functional significance of c-met in breast cancer: a review. Breast Cancer Res.

[143] Maroun CR, Rowlands T (2014). The met receptor tyrosine kinase: a key player in oncogenesis and drug resistance. Pharmacol Ther.

[144] Datta SR (1997). Akt phosphorylation of BAD couples survival signals to the cell-intrinsic death machinery. Cell.

[145] Hay N, Sonenberg N (2004). Upstream and downstream of mTOR. Genes Dev.

[146] Majumder PK (2004). mTOR inhibition reverses Akt-dependent prostate intraepithelial neoplasia through regulation of apoptotic and HIF-1-dependent pathways. Nat Med.

[147] Parkhitko AA (2014). Kinase mTOR: regulation and role in maintenance of cellular homeostasis, tumor development, and aging. Biochemistry (Mosc).

[148] Nagasawa T (2014). CXC chemokine ligand 12 (CXCL12) and its receptor CXCR4. J Mol Med (Berl).

[149] Smith MC (2004). CXCR4 regulates growth of both primary and metastatic breast cancer. Cancer Res.

[150] Phillips RJ (2005). Epidermal growth factor and hypoxia-induced expression of CXC chemokine receptor 4 on non-small cell lung cancer cells is regulated by the phosphatidylinositol 3-kinase/PTEN/AKT/mammalian target of rapamycin signaling pathway and activation of hypoxia inducible factor-1alpha. J Biol Chem.

[151] Cagnol S, Chambard JC (2010). ERK and cell death: mechanisms of ERK-induced cell death--apoptosis, autophagy and senescence. FEBS J.

[152] Dhillon AS (2007). MAP kinase signalling pathways in cancer. Oncogene.

[153] Li W, Melton DW (2012). Cisplatin regulates the MAPK kinase pathway to induce increased expression of DNA repair gene ERCC1 and increase melanoma chemoresistance. Oncogene.

[154] MacCorkle RA, Tan TH (2005). Mitogen-activated protein kinases in cell-cycle control. Cell Biochem Biophys.

[155] Zhang W, Liu HT (2002). MAPK signal pathways in the regulation of cell proliferation in mammalian cells. Cell Res.

[156] Arrighetti N (2016). PKC-alpha modulation by miR-483-3p in platinum-resistant ovarian carcinoma cells. Toxicol Appl Pharmacol.

[157] Basu A, Woolard MD, Johnson CL (2001). Involvement of protein kinase C-delta in DNA damage-induced apoptosis. Cell Death Differ.

[158] Yin J, et al. circ_C20orf11 enhances DDP resistance by inhibiting miR-527/YWHAZ through the promotion of extracellular vesicle-mediated macrophage M2 polarization in ovarian cancer. Cancer Biol Ther. 2021:1–15. 10.1080/15384047.2021.1959792 PMC8489911 34382916

[159] Abbaszadegan MR, Moghbeli M (2018). Role of MAML1 and MEIS1 in esophageal squamous cell carcinoma depth of invasion. Pathol Oncol Res.

[160] Abbaszadegan MR (2018). WNT and NOTCH signaling pathways as activators for epidermal growth factor receptor in esophageal squamous cell carcinoma. Cell Mol Biol Lett.

[161] Moghbeli M (2015). Role of Msi1 and MAML1 in regulation of Notch signaling pathway in patients with esophageal squamous cell carcinoma. J Gastrointest Cancer.

[162] Moghbeli M (2019). Role of MAML1 in targeted therapy against the esophageal cancer stem cells. J Transl Med.

[163] Liu YP (2013). Cisplatin selects for multidrug-resistant CD133+ cells in lung adenocarcinoma by activating Notch signaling. Cancer Res.

[164] McAuliffe SM (2012). Targeting Notch, a key pathway for ovarian cancer stem cells, sensitizes tumors to platinum therapy. Proc Natl Acad Sci U S A.

[165] Gao S (2017). PSAT1 is regulated by ATF4 and enhances cell proliferation via the GSK3beta/beta-catenin/cyclin D1 signaling pathway in ER-negative breast cancer. J Exp Clin Cancer Res.

[166] Liu H (2015). SOX9 overexpression promotes Glioma metastasis via Wnt/beta-catenin signaling. Cell Biochem Biophys.

[167] Santos JC (2016). SOX9 elevation acts with canonical WNT signaling to drive gastric cancer progression. Cancer Res.

[168] Speck NA, Gilliland DG (2002). Core-binding factors in haematopoiesis and leukaemia. Nat Rev Cancer.

[169] Xiao L (2020). Inhibition of RUNX1 promotes cisplatin-induced apoptosis in ovarian cancer cells. Biochem Pharmacol.

[170] Katoh M (2013). Cancer genetics and genomics of human FOX family genes. Cancer Lett.

[171] Aparicio IM (2016). The autophagy-related protein LC3 is processed in stallion spermatozoa during short-and long-term storage and the related stressful conditions. Animal.

[172] Ichimura Y (2008). Structural basis for sorting mechanism of p62 in selective autophagy. J Biol Chem.

[173] Valente G (2014). Expression and clinical significance of the autophagy proteins BECLIN 1 and LC3 in ovarian cancer. Biomed Res Int.

[174] Hu Z (2020). miR-29c-3p inhibits autophagy and cisplatin resistance in ovarian cancer by regulating FOXP1/ATG14 pathway. Cell Cycle.

[175] Tian M (2019). Modulation of Myb-induced NF-kB -STAT3 signaling and resulting cisplatin resistance in ovarian cancer by dietary factors. J Cell Physiol.

[176] Zhang Y (2007). Critical role of c-Jun overexpression in liver metastasis of human breast cancer xenograft model. BMC Cancer.

[177] Xue M, Chen W, Li X (2016). Urothelial cancer associated 1: a long noncoding RNA with a crucial role in cancer. J Cancer Res Clin Oncol.

[178] Niu G (2002). Constitutive Stat3 activity up-regulates VEGF expression and tumor angiogenesis. Oncogene.

[179] Jin P, Liu Y, Wang R. STAT3 regulated miR-216a promotes ovarian cancer proliferation and cisplatin resistance. Biosci Rep. 2018;38(4):BSR20180547. 10.1042/BSR20180547 PMC6131203 30061175

[180] Reimer D (2010). E2F3a is critically involved in epidermal growth factor receptor-directed proliferation in ovarian cancer. Cancer Res.

[181] Reimer D (2011). Regulation of transcription factor E2F3a and its clinical relevance in ovarian cancer. Oncogene.

[182] Nilsson JA, Cleveland JL (2003). Myc pathways provoking cell suicide and cancer. Oncogene.

[183] Prathapam T (2010). p27Kip1 mediates addiction of ovarian cancer cells to MYCC (c-MYC) and their dependence on MYC paralogs. J Biol Chem.

[184] Pyndiah S (2011). C-MYC suppresses BIN1 to release poly(ADP-ribose) polymerase 1: a mechanism by which cancer cells acquire cisplatin resistance. Sci Signal.

[185] Fischer KR (2015). Epithelial-to-mesenchymal transition is not required for lung metastasis but contributes to chemoresistance. Nature.

[186] Puisieux A, Brabletz T, Caramel J (2014). Oncogenic roles of EMT-inducing transcription factors. Nat Cell Biol.

[187] Qin Q (2012). Normal and disease-related biological functions of Twist1 and underlying molecular mechanisms. Cell Res.

[188] Cao R, Zhang Y (2004). The functions of E(Z)/EZH2-mediated methylation of lysine 27 in histone H3. Curr Opin Genet Dev.

[189] Guo J (2011). EZH2 regulates expression of p57 and contributes to progression of ovarian cancer in vitro and in vivo. Cancer Sci.

[190] Jacobs JJ, van Lohuizen M (2002). Polycomb repression: from cellular memory to cellular proliferation and cancer. Biochim Biophys Acta.

[191] Gupta RA (2010). Long non-coding RNA HOTAIR reprograms chromatin state to promote cancer metastasis. Nature.

[192] Rao PS (2009). RNF2 is the target for phosphorylation by the p38 MAPK and ERK signaling pathways. Proteomics.

[193] Xia Y, Jiang L, Zhong T (2018). The role of HIF-1alpha in chemo−/radioresistant tumors. Onco Targets Ther.

[194] Du J, Zhang L (2015). Integrated analysis of DNA methylation and microRNA regulation of the lung adenocarcinoma transcriptome. Oncol Rep.

[195] Suzuki H (2012). DNA methylation and microRNA dysregulation in cancer. Mol Oncol.

[196] Fumagalli C (2014). Prevalence and clinicopathologic correlates of O(6)-methylguanine-DNA methyltransferase methylation status in patients with triple-negative breast cancer treated preoperatively by alkylating drugs. Clin Breast Cancer.

[197] Kwon MJ, Shin YK (2011). Epigenetic regulation of cancer-associated genes in ovarian cancer. Int J Mol Sci.

[198] Ween MP (2015). The role of ABC transporters in ovarian cancer progression and chemoresistance. Crit Rev Oncol Hematol.

[199] Haenisch S, Werk AN, Cascorbi I (2014). MicroRNAs and their relevance to ABC transporters. Br J Clin Pharmacol.

[200] Popeda M, Pluciennik E, Bednarek AK (2014). Proteins in cancer multidrug resistance. Postepy Hig Med Dosw.

[201] Benyamin B (2014). Novel loci affecting iron homeostasis and their effects in individuals at risk for hemochromatosis. Nat Commun.

[202] Wu J (2017). Nrf2 induces cisplatin resistance via suppressing the iron export related gene SLC40A1 in ovarian cancer cells. Oncotarget.

[203] Sun S (2017). The association between copper transporters and the prognosis of cancer patients undergoing chemotherapy: a meta-analysis of literatures and datasets. Oncotarget.

[204] Kalayda GV (2008). Altered localisation of the copper efflux transporters ATP7A and ATP7B associated with cisplatin resistance in human ovarian carcinoma cells. BMC Cancer.

[205] Cabodi S (2010). Integrin signalling adaptors: not only figurants in the cancer story. Nat Rev Cancer.

[206] Wang WW (2015). Integrin beta-8 (ITGB8) silencing reverses gefitinib resistance of human hepatic cancer HepG2/G cell line. Int J Clin Exp Med.

[207] Oeggerli M (2012). Role of KCNMA1 in breast cancer. PLoS One.

[208] Sokolowski B (2011). Conserved BK channel-protein interactions reveal signals relevant to cell death and survival. PLoS One.

[209] Taguchi K, Yamamoto M (2017). The KEAP1-NRF2 system in cancer. Front Oncol.

